# Demographic and Support Interest Differences Among Nonbirthing Parents Using a Digital Health Platform With Parenthood-Related Anxiety: Cross-Sectional Study

**DOI:** 10.2196/46152

**Published:** 2023-11-20

**Authors:** Adam K Lewkowitz, Lily Rubin-Miller, Hannah R Jahnke, Melissa A Clark, Caron Zlotnick, Emily S Miller, Natalie Henrich

**Affiliations:** 1 Department of Obstetrics and Gynecology, Warren Alpert Medical School at Brown University, Providence, RI, United States; 2 Maven Clinic, New York, NY, United States; 3 Department of Health Services, Policy, & Practice, Brown School of Public Health, Providence, RI, United States; 4 Department of Psychiatry and Human Behavior, Warren Alpert Medical School at Brown University, Providence, RI, United States

**Keywords:** nonbirthing parent, paternal mental health, perinatal anxiety, parenting anxiety, digital health, anxiety, perinatal, mental health support, digital platform, pregnancy, parents, spouse, partners, support, support groups, online support

## Abstract

**Background:**

The transition to parenthood is a period of major stressors and increased risk of anxiety for all parents. Though rates of perinatal anxiety are similar among women (4%-25%) and men (3%-25%), perinatal anxiety research on nonbirthing partners remains limited.

**Objective:**

We aimed to examine whether demographic characteristics or digital perinatal support preferences differed among nonbirthing partners with compared to without self-reported high parenthood-related anxiety.

**Methods:**

In this large cross-sectional study of nonbirthing partners using a digital perinatal health platform during their partner’s pregnancy, users reported their parenthood-related anxiety through a 5-item Likert scale in response to the prompt “On a scale of 1=None to 5=Extremely, how anxious are you feeling about parenthood?” High parenthood-related anxiety was defined as reporting being very or extremely anxious about parenthood. During the onboarding survey, in response to the question “Which areas are you most interested in receiving support in?” users selected as many support interests as they desired from a list of options. Chi-square and Fisher exact tests were used to compare demographic characteristics and support interests of nonbirthing partners with low versus high parenthood anxiety. Logistic regression models estimated the odds ratios (ORs), with 95% CIs, of high parenthood-related anxiety with each user characteristic or digital support interest.

**Results:**

Among 2756 nonbirthing partners enrolled in the digital platform during their partner’s pregnancy, 2483 (90.1%) were men, 1668 (71.9%) were first-time parents, 1159 (42.1%) were non-Hispanic White, and 1652 (50.9%) endorsed an annual household income of >US $100,000. Overall, 2505 (91.9%) reported some amount of parenthood-related anxiety, and 437 (15.9%) had high parenthood-related anxiety. High parenthood-related anxiety was more common among non-White nonbirthing partners: compared to those who identified as non-Hispanic White, those who identified as Asian, Black, or Hispanic had 2.39 (95% CI 1.85-3.08), 2.01 (95% CI 1.20-3.23), and 1.68 (95% CI 1.15-2.41) times the odds of high parenthood-related anxiety, respectively. Lower household income was associated with increased odds of reporting high parenthood anxiety, with the greatest effect among those with annual incomes of <US $50,000 compared to >US $100,000 (OR 2.13, 95% CI 1.32-3.34). In general, nonbirthing partners were interested in receiving digital support during their partner’s pregnancy, but those with high parenthood-related anxiety were more likely to desire digital support for all support interests compared to those without high parenthood anxiety. Those with high parenthood-related anxiety had more than 2 times higher odds of requesting digital education about their emotional health compared to those without high parenthood-related anxiety (OR 2.06, 95% CI 1.67-2.55).

**Conclusions:**

These findings demonstrate the need for perinatal anxiety-related support for all nonbirthing partners and identify nonbirthing partners’ demographic characteristics that increase the odds of endorsing high parenthood-related anxiety. Additionally, these findings suggest that most nonbirthing partners using a digital health platform with high parenthood-related anxiety desire to receive perinatal mental health support.

## Introduction

The transition to parenthood is a period of major stressors and increased risk of mental health issues, regardless of whether or not the parent gives birth [1-4]. Indeed, when stratified by gender (which frequently corresponds to birthing and nonbirthing roles), rates of perinatal anxiety are similar among women (4%-25%) [1,2] and men (3%-25%) [3,4]. Despite the similar burden of perinatal anxiety between parents and the known interplay between maternal and paternal perinatal mood disorders [5-7], perinatal anxiety research on nonbirthing partners remains limited, and little is known about the desire of nonbirthing partners to receive mental health support during the perinatal period [8]. Thus, we aimed to examine whether demographic characteristics or desired mental health supports differed among nonbirthing partners with compared to without high parenthood-related anxiety.

## Methods

### Eligibility Criteria/Recruitment

This study examined a cohort of users enrolled in the partner pathway in Maven, a digital health platform for pregnant people and their partners, from March 16, 2021, through October 20, 2022. Access to Maven is a sponsored benefit through an employer or health plan of the user or their partner. Users consented to the use of their deidentified data for scientific research upon creating an account on the digital platform. As previously described [9], users self-reported demographic information, desired support interests, and parenthood-related anxiety on a health survey at onboarding. For race and ethnicity, users selected a single option from a list of choices. In response to the question “Which areas are you most interested in receiving support in?” users selected as many support interests as they desired from the following list: “choosing a healthcare provider/team,” “labor and delivery options,” “preparing to be a working parent,” “infant care,” “learning about childcare options,” “my own emotional health,” “understanding my partner’s physical experience during pregnancy,” and “understanding my partner’s emotional experience during pregnancy.” Users reported their parenthood-related anxiety on a 5-item Likert scale in response to the prompt “On a scale of 1=None to 5=Extremely, how anxious are you feeling about parenthood?” To be included, users had to be enrolled in Maven’s partner track and have completed the Maven Clinic’s onboarding survey while their partner was pregnant. Thus, data for these analyses included platform utilization and user-reported data from the onboarding questionnaire.

### Statistical Analysis

Due to small sample sizes, participants who reported their race/ethnicity as American Indian or Alaskan Native, Native Hawaiian or other Pacific Islander, or multiple races were categorized in the “other” category. Income was assessed categorically. High parenthood-related anxiety was defined as responding to the question on parenthood-related anxiety with a 4 (“very”) or 5 (“extremely”). Some parenthood-related anxiety was defined as responding to the question on parenthood-related anxiety with any response other than a 1 (“none”) on the Likert scale. Descriptive analyses assessed user demographics and support interests stratified by presence of high parenthood-related anxiety. In bivariate analyses, the chi-square or Fisher exact tests were used to assess categorical variables. Logistic regression models estimated the odds ratio (ORs) and 95% CI of reporting high parenthood anxiety with each user characteristic or educational support preference. All analyses were conducted in R (version 3.6.3; R Foundation for Statistical Computing).

### Ethical Considerations

The study was designated as exempt by the WCG Institutional Review Board, an independent ethical review board.

## Results

Of the 4188 users enrolled in Maven’s partner pathway during the study period, 3705 (88.5%) completed the onboarding survey. Of these, 2756 (74.4%) completed their survey while their partner was pregnant and were included for analysis. Overall, most (n=2483, 90.1%) nonbirthing partners self-identified as male, 2034 (73.8%) identified as first-time parents, and 2505 (91.9%) nonbirthing partners endorsed feeling at least some parenthood-related anxiety.

In this study population, 437 (15.9%) of nonbirthing partners were categorized as having high parenthood-related anxiety. Some demographic characteristics increased the odds of endorsing high parenthood-related anxiety (Table 1). Specifically, compared to non-Hispanic White nonbirthing partners, the odds of participants reporting high parenthood-related anxiety were more than 2-fold higher among Asian (OR 2.39, 95% CI 1.85-3.08) or Black (OR 2.01, 95% CI 1.20-3.23) nonbirthing partners and more than 60% higher among Hispanic nonbirthing partners (OR 1.68, 95% CI 1.15-2.41). Similarly, compared to non–first-time parents, first-time nonbirthing partners had twice the odds of reporting high parenthood-related anxiety (OR 2.01, 95% CI 1.55-2.65), and those with annual incomes of <US $50,000 or between US $50,000 and US $100,000 had more than 2-fold or 48% increased odds, respectively, of endorsing high parenthood-related anxiety compared to those with annual incomes of ≥US $100,000 (<US $50,000: OR 2.13, 95% CI 1.32-3.34; US $50,000-US $100,000: OR 1.48, 95% CI 1.07-2.01). The odds of endorsing high parenthood-related anxiety were similar between male and female nonbirthing parents (OR 1.20, 95% CI 0.69-1.98).

**Table 1.** Nonbirthing partner characteristics and support interests by parenthood-related anxiety level.

Nonbirthing partner characteristics		Overall (n=2756), n (%)	Low parenthood-related anxiety (n=2319, 84.1%), n (%)	High parenthood-related anxiety (n=437, 15.9%), n (%)	Odds ratio (95% CI)	*P* value
**Race/ethnicity**						
	Non-Hispanic White	1159 (42.1)	1033 (44.5)	126 (28.8)	Reference	N/A^a^
	Asian	714 (25.9)	553 (23.8)	161 (36.8)	2.39 (1.85-3.08)	<.001
	Hispanic	265 (9.6)	220 (9.5)	45 (10.3)	1.68 (1.15-2.41)	.006
	Black	117 (4.2)	94 (4.1)	23 (5.3)	2.01 (1.20-3.23)	.006
	Other	501 (18.2)	419 (18.1)	82 (18.8)	1.60 (1.19-2.16)	.002
**First child**						
	No	722 (26.2)	651 (28.1)	71 (16.2)	Reference	N/A
	Yes	2034 (73.8)	1668 (71.9)	366 (83.8)	2.01 (1.55-2.65)	<.001
**Biological sex**						.84
	Female	98 (3.6)	80 (3.4)	18 (4.1)	1.20 (0.69-1.98)	.50
	Male	2483 (90.1)	2091 (90.2)	392 (89.7)	Reference	N/A
	Missing	175 (6.3)	148 (6.4)	27 (6.2)	N/A	N/A
**Household income (US $)**						.002
	Less than $50,000	103 (3.7)	76 (3.3)	27 (6.2)	2.13 (1.32-3.34)	.001
	$50,000-$100,000	299 (10.8)	240 (10.3)	59 (13.5)	1.48 (1.07-2.01)	.02
	More than $100,000	1652 (59.9)	1416 (61.1)	236 (54)	Reference	N/A
	Did not disclose	702 (25.5)	587 (25.3)	115 (26.3)	N/A	N/A
**Support interests**						
	Leaning about child care options	1403 (50.9)	1159 (50)	244 (55.8)	1.28 (1.04-1.59)	.02
	Choosing a health care provider/team	789 (28.6)	632 (27.3)	157 (35.9)	1.51 (1.21-1.88)	<.001
	My own emotional health	1032 (37.4)	806 (34.8)	226 (51.7)	2.06 (1.67-2.55)	<.001
	Infant care	2016 (73.1)	1674 (72.2)	342 (78.3)	1.47 (1.13-1.92)	.005
	Labor and delivery options	1200 (43.5)	969 (41.8)	231 (52.9)	1.60 (1.29-1.97)	<.001
	Understanding my partner’s emotional experience during pregnancy	1981 (71.9)	1635 (70.5)	346 (79.2)	1.72 (1.32-2.26)	<.001
	Understanding my partner’s physical experience during pregnancy	1825 (66.2)	1495 (64.5)	330 (75.5)	1.82 (1.42-2.34)	<.001
	Preparing to be a working parent	1779 (64.6)	1469 (63.3)	310 (70.9)	1.47 (1.16-1.86)	.001

a N/A: not applicable.

In general, nonbirthing partners were interested in receiving digital support during their partner’s pregnancy: the most requested support interests overall were infant care and understanding their partner’s emotional experience during pregnancy (Table 1). However, those with high parenthood anxiety were more likely to desire digital support from all support interests compared to those without high parenthood anxiety. In particular, the odds of nonbirthing partners desiring to learn more about their own emotional health or their partner’s physical or emotional experience during pregnancy were markedly higher compared to those without high parenthood-related anxiety (own emotional health: OR 2.06, 95% CI 1.67-2.55; partner’s physical experience in pregnancy: OR 1.82, 95% CI 1.42-2.34; partner’s emotional experience in pregnancy: OR 1.72, 95% CI 1.32-2.26).

## Discussion

### Principal Results

In this large sample, nearly all nonbirthing partners reported feeling at least some parenthood-related anxiety, and a substantial proportion of nonbirthing partners desired education about their own or their partner’s emotional health during the perinatal period. These findings demonstrate the need for perinatal mental health support for all parents, not just those who give birth, and suggest that digital health platforms may serve as logical entry points for nonbirthing partners to receive perinatal mental health support, as has been proposed [10]. Furthermore, demographic factors such as identifying as non-White or having an annual income of less than US $50,000 were associated with increased odds of high parenthood-related anxiety. This suggests that some nonbirthing partners may face a disproportionate burden of parenthood-related anxiety, and perinatal support interventions targeting these subpopulations may improve equity for nonbirthing partners.

### Comparisons With Prior Work

The rate of high parenthood-related anxiety in our study is consistent with levels of paternal anxiety in the literature (3%-25%) [3,4]. Furthermore, the high prevalence of any self-reported anxiety in our study population supports prior findings suggesting that the most common mental health diagnosis for nonbirthing partners in the perinatal period is adjustment disorder with anxiety symptoms [11].

### Clinical and Research Implications

In the United States, most current perinatal care delivery models do not provide much perinatal education and mental health support to nonbirthing partners [12]. Furthermore, nonbirthing partners are known to have lower rates of engagement with health care than birthing partners [12,13]. In this study, nonbirthing partners who were participating in a digital perinatal health platform not only actively selected the perinatal educational support topics about which they wanted to learn but voluntarily endorsed the presence and extent of their parenthood-related anxiety. These findings suggest that digitally screening nonbirthing partners during their partner’s pregnancy for their perinatal mental health and pregnancy-related health education needs could fill an important gap in nonbirthing partners’ perinatal experience. Mental health support and educational content could be delivered digitally or via in-person education provided from prenatal care providers when nonbirthing partners present to prenatal care visits, as has been proposed [10,14]. However, prior to widespread transformation of prenatal care delivery models, more research is needed to determine the optimal way to screen nonbirthing partners for mental health needs or perinatal educational preferences and to provide requested education and support based on the results of this screening.

### Limitations

Despite the large study population, our study has limitations. Parenthood-related anxiety was self-reported, rather than identified via a validated anxiety measure. There is also a risk of selection bias since all participants in this study were already actively engaged in a digital health platform. Additionally, the cross-sectional design of this study limits causal interpretation of our results. Furthermore, because outcomes were not adjusted for confounders, some bias may remain in our unadjusted analyses. Lastly, though our study population was large, generalizability of our findings may be reduced as many participants reported annual incomes of >US $100,000 and people of some races/ethnicities were overly represented in the sample.

### Conclusions

Among nonbirthing partners who used a digital health platform, most had some parenthood-related anxiety, and the odds of endorsing high parenthood-related anxiety were increased among non-White birthing partners, as well as those with annual incomes of less than US $100,000 and, in particular, less than US $50,000. Furthermore, though nonbirthing partners desired education on their own or their partner’s emotional health during the perinatal period, the odds of desiring perinatal mental health support were higher among nonbirthing partners who endorsed high parenthood-related anxiety. These findings demonstrate not only the need for perinatal anxiety-related support for all nonbirthing partners, but that most nonbirthing partners with high parenthood-related anxiety using a digital perinatal platform desire to receive digital perinatal mental health support.

## Abbreviations

OR: odds ratio
